# Empagliflozin Ameliorates Diabetic Cardiomyopathy by Inhibiting Ferroptosis via SIRT3: Mechanisms and Therapeutic Implications

**DOI:** 10.3390/antiox15050543

**Published:** 2026-04-24

**Authors:** Taoshan Feng, Meilian Liu, Dan Zhong, Xusan Xu, Zhengqiang Luo, Wensen Zhang, Yajun Wang, Riling Chen, Xiaoming Chen, Guoda Ma

**Affiliations:** 1 Maternal and Child Research Institute, Shunde Women and Children Hospital, Guangdong Medical University, Foshan 528300, China; taoshanfeng@gdmu.edu.cn (T.F.); danzhong@gdmu.edu.cn (D.Z.); xuxusan158806046@163.com (X.X.); luozhengqiang0055@163.com (Z.L.); 13760679221@163.com (W.Z.); yajunw@gdmu.edu.cn (Y.W.); chenrl319@163.com (R.C.); 2 Department of Endocrinology, The Second Affiliated Hospital of Guangdong Medical University, Zhanjiang 524000, China; 3 Department of Pulmonary Oncology, Affiliated Hospital of Guangdong Medical University, Zhanjiang 524000, China; liumeilian@gdmu.edu.cn; 4 Faculty of Chinese Medicine, Macau University of Science and Technology, Macau 999078, China

**Keywords:** diabetic cardiomyopathy, empagliflozin, SIRT3, ferroptosis

## Abstract

Empagliflozin (EMPA), a sodium-glucose cotransporter 2 inhibitor, has garnered attention for its cardiovascular benefits beyond glycemic control. Ferroptosis, a novel form of regulated cell death, contributes to the pathogenesis of diabetic cardiomyopathy (DCM). However, whether EMPA mitigates DCM by suppressing ferroptosis remains unclear. Here, Type 2 diabetic db/db mice were used to establish a DCM model and treated with EMPA (10 mg/kg/day) for 12 weeks. EMPA significantly improved cardiac function, reduced myocardial fibrosis, and attenuated ferroptosis, concomitant with upregulated silent information regulator 3 (SIRT3) expression. In the rat cardiomyocytes (H9c2 cells) exposed to high glucose and palmitic acid, EMPA treatment or SIRT3 overexpression alleviated oxidative stress, mitochondrial dysfunction, and ferroptosis. Mechanistically, molecular docking, molecular dynamics simulation, cellular thermal shift assay and drug affinity responsive target stability assay confirmed that SIRT3 is the drug target of EMPA, stabilizing its protein levels and reducing acetylated p53 expression. Notably, SIRT3 silencing abolished EMPA’s beneficial effects on oxidative stress and ferroptosis. Our findings demonstrate that EMPA exerts cardioprotective effects by inhibiting oxidative stress and ferroptosis in cardiomyocytes, which is mediated by SIRT3. This study provides novel insights into the mechanisms underlying EMPA’s therapeutic effects in DCM.

## 1. Introduction

Diabetes mellitus (DM) is a metabolic disorder characterized by insufficient insulin secretion or insulin resistance, leading to hyperglycemia and elevated glycated hemoglobin levels [1,2]. Chronic metabolic abnormalities, persistent hyperglycemia, and insulin resistance significantly increase the risk of cardiovascular mortality in diabetic patients, which is 2.5 times higher than in non-diabetic individuals [3]. Diabetic cardiomyopathy (DCM), also termed non-vascular myocardial dysfunction, refers to a pathological process where diabetic patients develop myocardial structural abnormalities and clinical manifestations in the absence of other cardiac risk factors such as coronary artery disease, hypertension, or severe valvular disease [4]. Epidemiological studies indicate that DCM remains a major contributor to increased mortality among diabetic patients. With the rising prevalence of obesity and type 2 diabetes mellitus (T2DM), the incidence of DCM continues to escalate, imposing a substantial burden on global healthcare systems and economies [5].

The pathogenesis of DCM is complex and not yet fully understood. Ensuring sufficient survival of cardiomyocytes, mitigating myocardial fibrosis, and improving both diastolic and systolic cardiac function are crucial for alleviating and treating DCM [6]. Adult mammalian cardiomyocytes have extremely limited regenerative capacity [7], with diabetic cardiomyocytes exhibiting 85-fold higher mortality rates [8]. While apoptosis, necroptosis, autophagy, and pyroptosis in DCM have been extensively studied [9,10,11], the role of ferroptosis—an iron-dependent cell death characterized by lipid peroxidation [12]—remains insufficiently investigated [13,14]. First reported in cardiac disease in 2018 [15], ferroptosis may be particularly relevant in DCM given the heart’s high mitochondrial content and iron requirements [16]. Iron overload disrupts cellular homeostasis, generating excessive reactive oxygen species (ROS) and promoting mitochondrial damage [17]. Diabetic patients often exhibit iron overload, exacerbating insulin resistance and cardiovascular complications [18,19,20], with ROS accumulation further promoting ferroptosis [21].

Silent information regulator 3 (SIRT3), a major mitochondrial NAD^+^-dependent deacetylase regulating apoptosis, inflammation, and energy metabolism [22], plays crucial roles in DCM by maintaining mitochondrial function and reducing ROS [23]. While SIRT3 activation improves cardiac function in DCM models [24], the relationship between SIRT3 and ferroptosis in DCM remains unclear.

Empagliflozin (EMPA), a sodium-glucose cotransporter 2 (SGLT2) inhibitor, is one of the most widely studied agents in its class, with demonstrated cardiovascular benefits in the large clinical trial EMPA-REG OUTCOME [25,26]. Recent evidence has demonstrated SGLT2 expression in human cardiomyocytes [27,28], supporting the possibility that EMPA may act directly on these cells via SGLT2. Emerging evidence suggests that EMPA may regulate ferroptosis and restore SIRT3 expression [29,30], and it has been shown to improve DCM through multiple pathways [31,32,33,34]. Given its well-established clinical profile, EMPA serves as an ideal candidate to explore the mechanistic link between SGLT2 inhibition, SIRT3, and ferroptosis in DCM. However, whether EMPA ameliorates DCM through SIRT3-mediated ferroptosis inhibition remains unknown. This study investigates this novel mechanism using db/db mice and H9c2 cell models, aiming to provide new therapeutic targets for DCM treatment.

## 2. Materials and Methods

### 2.1. Animals

Eight-week-old male db/db mice (leptin receptor-deficient, diabetic) and db/m mice (heterozygous lean controls), purchased from Huachuang Sino Pharma Tech Co., Ltd. (Taizhou, Jiangsu, China), were kept at room temperature (~23 °C) under a standard 12:12 h (hours) light/dark cycle. All db/db mice exhibited fasting blood glucose (FBG) levels >16.7 mmol/L after a 12 h fast (tail vein). Male db/m mice were used as a control group. After 2–3 days of acclimation, intragastric gavage administration was carried out with conscious animals, using straight gavage needles appropriate for the animal size. All animals were anesthetized by intraperitoneal injection of 1.25% avidin (20 mL/kg) to collect tissues. The mice were divided into three groups with 5 mice in each group (15 mice in total): (1) Control group (CON); (2) db/db mice group (DM); and (3) EMPA-treated db/db mice group (DM + EMPA). In the DM + EMPA group, EMPA (Boehringer Ingelheim, Ingelheim, Germany) was administrated via oral gavage at a dose of 10 mg/kg/d for 12 weeks (using 0.5% hydroxyethyl cellulose as the vehicle) [35,36]. Both CON and DM groups were gavaged with 0.5% hydroxyethyl cellulose vehicle following the same dosing regimen.

All experiments conformed to the recommended guidelines for animal experimentation and were approved by the Animal Research Ethics Committee of Guangdong Medical University (AHGDMU-LAC-A-202407-230). All appropriate measures were taken to minimize the pain or discomfort of animals.

### 2.2. Intraperitoneal Glucose Tolerance Test (IPGTT) and Intraperitoneal Insulin Tolerance Test (ITT)

Mice were fasted overnight and intraperitoneally injected with 1 g/kg glucose for IPGTT. For ITT, mice were fasted for 6 h and injected intraperitoneally with 0.5 U/kg insulin. Blood glucose levels were measured from the tail vein at 0 (before glucose administration), 15, 30, 60, and 120 min.

### 2.3. Echocardiography

Cardiac function was assessed using the Vevo 2100 ultrasound system (Visual Sonics, Toronto, ON, Canada). Mice were anesthetized with 2% isoflurane and maintained at 37 °C in supine position. M-mode measurements were obtained from the parasternal long-axis view, averaging at least two cardiac cycles. Ejection fraction (EF) and fractional shortening (FS) were calculated to evaluate systolic function.

### 2.4. Histological Evaluation

Left ventricular myocardium was rinsed with ice-cold phosphate-buffered saline (PBS) and fixed in 4% paraformaldehyde. After paraffin embedding, 5 μm sections were prepared for staining. Hematoxylin and eosin (HE) and wheat germ agglutinin (WGA) staining were performed to evaluate myocardial hypertrophy, while Masson’s trichrome staining assessed fibrosis. Collagen deposition was quantified as the fibrotic area percentage using the ImageJ 1.53 software (National Institutes of Health, Bethesda, MD, USA), with six randomly selected fields (400× magnification) analyzed per sample.

### 2.5. Prussian Blue Staining

Iron deposition was assessed by Prussian Blue staining (Solarbio, Beijing, China). Briefly, dewaxed heart sections were incubated with freshly prepared potassium ferrocyanide/HCl solution (1:1) for 45 min, rinsed, counterstained with eosin, and mounted. Iron-positive areas (blue) were quantified in five random fields/section using ImageJ (400×).

### 2.6. Transmission Electron Microscopy (TEM)

Mitochondrial ultrastructure was examined by TEM (Hitachi H-7500, Tokyo, Japan). Left ventricular tissues were fixed in 2.5% glutaraldehyde (Servicebio, Wuhan, China), followed by standard dehydration and epoxy resin embedding. Ultrathin sections (80 nm) were prepared using glass knives on an ultramicrotome for observation of myofilaments and mitochondria.

### 2.7. Measurement of Serum Creatine Kinase-MB (CK-MB)

Blood samples were collected via retro-orbital bleeding, which was centrifuged at 1200 rpm for 15 min to obtain serum. The CK-MB was detected by the assay kit (Rayto, Shenzhen, China).

### 2.8. In Vitro Experiments

The immortalized rat cardiomyocyte cell line (H9c2) was purchased from the American Type Culture Collection (ATCC, Manassas, VA, USA) and cultured in Dulbecco’s Modified Eagle Medium (Gibco, Grand Island, NY, USA) containing 5.5 mmol/L glucose, supplemented with 10% fetal bovine serum (Vivacell, Shanghai, China) and 100 U/mL penicillin/streptomycin (Gibco, Grand Island, NY, USA) in a humidified incubator with 5% CO_2_ at 37 °C. According to the literature [13,37], in this research, the DCM cell model (HG/PA group) was constructed by 33.3 mM glucose and 200 μM palmitic acid (PA). Cells were divided into four groups: control (CON group), EMPA treated (EMPA group), high glucose and PA co-treatment (HG/PA group), and HG/PA and EMPA combined treatment (HG/PA + EMPA group). H9c2 cells were cultured with DMEM containing glucose, PA and a final concentration of 1 μM EMPA (MedChemExpress, Monmouth Junction, NJ, USA). After 24 h of treatment, the H9c2 cells in each group were measured for related indexes. To explore the occurrence of ferroptosis in H9c2 cells treated with HG/PA, pretreatment with Ferrostatin-1 (Fer-1, 10 μM)—a ferroptosis inhibitor—for 2 h was required.

### 2.9. Transfections Experiments

Lipofectamine 3000 (Invitrogen, Carlsbad, CA, USA) was used for transient transfection of Vector (pLV-EGFP) and SIRT3-overexpression plasmid (pLV-rSIRT3; Yunzhou Biosciences Co., Ltd., Guangzhou, China) into H9c2 cells, following the manufacturer’s protocol. The recombinant plasmid pLV-rSIRT3 information is shown in Figure S1. To silence SIRT3, SIRT3 siRNA was purchased from Ribobio (Guangzhou, China). The sequence of SIRT3 (rat) siRNAs is: 5ʹ-CCAAUGUCGCUCACUACUU-3ʹ. The H9c2 cells were transfected with SIRT3 siRNA (80 nM) and negative control (NC, 80 nM) for 48 h using Lipofectamine 3000 (Invitrogen, Carlsbad, CA, USA), respectively.

### 2.10. Cell Viability and Mortality Determination

Cell viability was assessed using CCK-8 kit (GlpBio, Montclair, CA, USA). H9c2 cells (70–80% confluent) in 96-well plates were treated with EMPA for 24 h, followed by 10 μL CCK-8 incubation (60 min, 37 °C) and absorbance measurement at 450 nm. Cell death was evaluated by Calcein-AM/PI staining (Solarbio, Beijing, China). Briefly, H9c2 cells (5 × 10^4^ cells/well) seeded in 15 mm confocal dishes were treated for 24 h, centrifuged (1000× *g*, 15 min), and incubated with 1 μL Calcein-AM and 1 μL PI in 1 mL buffer for 30 min at 37 °C in the dark. After washing with PBS, images were captured using a confocal microscope (488 nm for Calcein-AM, green; 535 nm for PI, red). At least 3–5 fields per group were acquired. Live (green) and dead (red) cells were quantified using ImageJ, and the mortality rate was calculated as: Dead cells (%) = [Red/(Red + Green)] × 100%.

### 2.11. Determination of ROS and Mitochondrial Reactive Oxygen Species (mitoROS) Generation

To quantify the levels of ROS and mitoROS in H9c2 cells, the cells were treated as indicated and then incubated with 10 μM dihydroethidium (DHE, APPLYGEN, Beijing, China) or 1 μM MitoSOX Red (Thermo Fisher Scientific, Waltham, MA, USA) for 30 min at 37 °C in the dark. Red fluorescence was detected using a laser scanning confocal microscope (Carl Zeiss AG, Oberkochen, Germany). The fluorescent intensity in different groups was determined by ImageJ.

### 2.12. Intracellular Fe^2+^ and Total Iron Assay

Cellular Fe^2+^ levels were detected using FerroOrange (1 μM, 30 min incubation in dark) (Dojindo, Kumamoto, Japan). Fluorescence imaging was performed at 555 nm excitation using a laser confocal microscope (Carl Zeiss AG, Oberkochen, Germany), with intensity quantification by ImageJ (minimum six random 400× fields per sample). Total iron content in cell lysates and myocardial tissues was measured using a commercial assay kit (Jiancheng Bioengineering Institute, Nanjing, China).

### 2.13. Detection of Malondialdehyde (MDA), Superoxide Dismutase (SOD), Glutathione/Glutathione Oxidized (GSH/GSSG) Ratio and Adenosine Triphosphate (ATP)

The relative concentration of MDA, the activity of SOD and GSH/GSSG ratio in H9c2 cells and myocardial tissue were assessed by using the corresponding commercial kits (Beyotime, Shanghai, China). The level of ATP in H9c2 cells was measured using an ATP assay kit (Beyotime, Shanghai, China).

### 2.14. Detection of Lipid Peroxidation Level

Lipid peroxidation levels were determined using C11-BODIPY 581/591 dye (5 μM; Thermo Fisher Scientific, Waltham, MA, USA). The reduced probe emits red fluorescence, while the oxidized form (oxC11-BODIPY) emits green fluorescence. After 30 min incubation at 37 °C, fluorescence signals were detected by confocal microscopy (Carl Zeiss AG, Oberkochen, Germany). A decrease in the red/green ratio indicates elevated lipid peroxidation.

### 2.15. Detection of Mitochondrial Membrane Potential (MMP)

MMP was measured using a JC-1 assay kit (Beyotime, Shanghai, China). The H9c2 cells were stained with JC-1 dye for 25 min at 37 °C; after washing with dilution buffer three times, the cells were imaged under a laser scanning confocal microscope (Carl Zeiss AG, Oberkochen, Germany). JC-1 forms red-fluorescent aggregates in healthy mitochondria with high membrane potential, but remains as green-fluorescent monomers upon depolarization. The red/green fluorescence ratio (aggregates/monomers) was calculated using ImageJ to evaluate MMP.

### 2.16. Western Blot

The heart and H9c2 cell proteins were lysed at 4 °C with radioimmunoprecipitation assay (RIPA) buffer (Beyotime, Shanghai, China) containing protease, phosphatase inhibitors and phenylmethanesulfonyl fluoride (1 mM, Beyotime, Shanghai, China). Protein concentration was determined using the BCA Protein Assay Kit (Beyotime, Shanghai, China). Samples were transferred to polyvinylidene fluoride membranes after 10% sodium dodecyl sulfate-polyacrylamide gel electrophoresis. Blocked in 5% skim milk for 1 h and incubated with primary antibodies: SIRT3 (1:1000, Cell Signaling Technology, Danvers, MA, USA), Collagen III (1:1000, Abcam, Cambridge, UK), p53 (1:1000, Proteintech, Rosemont, IL, USA), ac-p53 (1:1000, Beyotime, Shanghai, China), glutathione Peroxidase 4 (GPX4, 1:200, SANTA, Dallas, TX, USA), ferritin heavy chain 1 (FTH1, 1:1000, Cell Signaling Technology, Danvers, MA, USA), acyl-coA synthetase long-chain family member 4 (ACSL4, 1:200, SANTA, Dallas, TX, USA), transferrin receptor (TFR, 1:1000, Abcam, Cambridge, UK), Tubulin (1:1000, Proteintech, Rosemont, IL, USA), β-actin (1:1000, Proteintech, Rosemont, IL, USA), GAPDH (1:1000, Affinity, Cincinnati, OH, USA). Protein bands were measured using an ECL kit (picogram) (Affinity, Cincinnati, OH, USA) after combining with horseradish peroxidase-linked antibodies for 1 h.

### 2.17. Quantitative Real-Time PCR (qPCR)

Total RNA was isolated from the hearts of mice or H9c2 cells using TransZol reagent (Transgen, Beijing, China). cDNA was synthesized from 1 μg RNA using HiScript II Q RT SuperMix (Vazyme, Nanjing, China) in 20 μL reactions. Total DNA was extracted using the TIANamp Genomic DNA Kit (TIANGEN, Beijing, China). qPCR was performed using ChamQ SYBR Master Mix (Vazyme, Nanjing, China). For mitochondrial DNA (mtDNA) copy number determination, mitochondrial-encoded genes Cytochrome c Oxidase Subunit II (COX II) and NADH Dehydrogenase 1 (ND-1) were amplified by qPCR and normalized to nuclear-encoded genes Ribosomal Protein S18 (Rps18) and β-actin. The primers used for prostaglandin-endoperoxide synthase 2 (Ptgs2), Sirt3, and mtDNA-related genes are listed in Table S1 and Table S2, respectively. The relative gene expression levels were calculated using the 2^−ΔΔCT^ method. Each experiment was performed in triplicate.

### 2.18. Molecular Docking and Molecular Dynamics Simulations

Molecular docking analysis was conducted using AutoDock Vina 1.2.2 to investigate EMPA-SIRT3 interactions. The EMPA structure was retrieved from PubChem (https://pubchem.ncbi.nlm.nih.gov/), while the SIRT3 crystal structure (PDB ID: 3GLS, 2.5 Å resolution) was obtained from the Protein Data Bank (http://www.rcsb.org/). Protein and ligand preparations included water removal, polar hydrogen addition, and PDBQT format conversion. Docking parameters included a 30 Å × 30 Å × 30 Å grid box with 0.05 nm spacing to ensure comprehensive ligand sampling.

Molecular dynamics simulations were performed using GROMACS 2019. The system underwent 50,000-step energy minimization at 300 K, followed by equilibration in both canonical and isothermal-isobaric ensembles. System stability was assessed through 100 ns simulations, with root mean square deviation (RMSD) analysis of protein residue atomic positions.

### 2.19. Cellular Thermal Shift Assay (CETSA)

After a 12 h treatment with EMPA (1 μM), H9c2 cells were collected and resuspended in PBS. Aliquots (0.2 mL) were heated at designated temperatures for 3 min, subjected to two freeze–thaw cycles in liquid nitrogen, then centrifuged at 12,000 rpm for 15 min. The resulting supernatants were analyzed by Western blot.

### 2.20. Drug Affinity Responsive Target Stability (DARTS)

H9c2 cells were washed with PBS and lysed in RIPA buffer. Cell lysates were aliquoted and incubated with DMSO or different concentrations of EMPA (30 min, room temperature). Proteolysis was performed using 0.5 μg/μL Pronase E (MedChemExpress, Monmouth Junction, NJ, USA) for 30 min before terminating the reaction with protein loading buffer for further analysis.

### 2.21. Statistical Analysis

Data are presented as mean ± standard deviation (SD). GraphPad Prism 9 (San Diego, CA, USA) was used for mapping and analyses. Student’s *t*-test was used to compare the data from two groups, and one-way analysis of variance (ANOVA) with Bonferroni’s post hoc test was used to compare the data from three or more groups, after checking for normality (Kolmogorov–Smirnov) and homogeneity (Levene). Statistical significance was set at *p* < 0.05.

## 3. Results

### 3.1. Empagliflozin Ameliorates Diabetic Symptoms and Cardiac Injury in DCM

**Figure 1:**
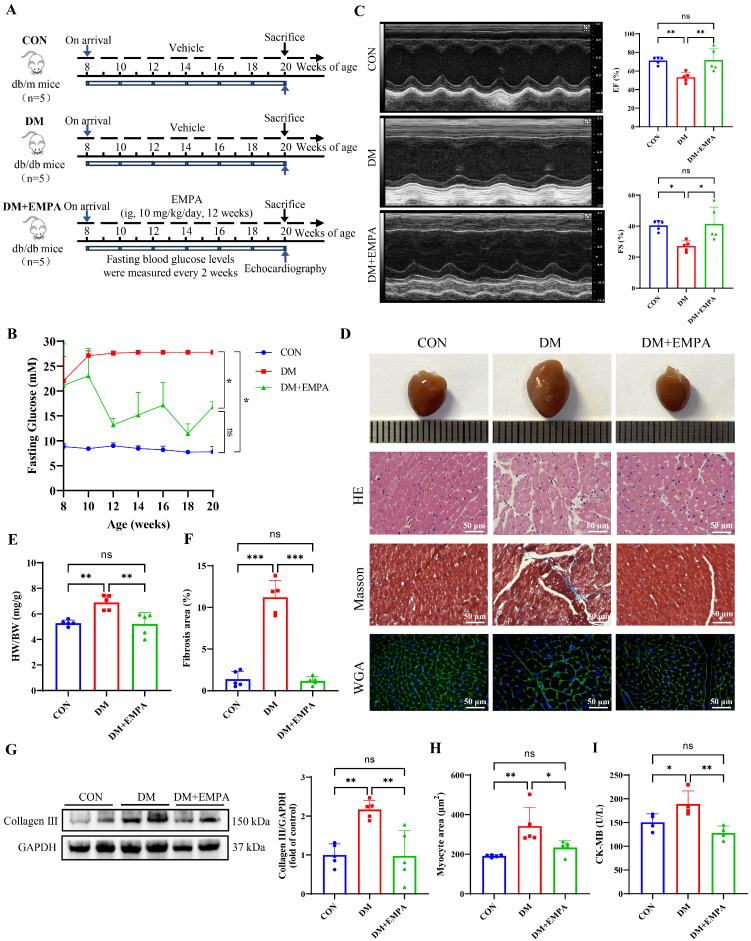
Empagliflozin improves the levels of blood glucose and cardiac injury in diabetic mice. (**A**) A schematic diagram showing the treatments of the mice. (**B**) FBG. (**C**) Representative M-mode echocardiography and quantification of EF and FS. (**D**) Representative gross heart images and histological staining including HE, Masson’s trichrome for fibrosis, and WGA for cardiomyocyte membrane outlining. (Scale bar = 50 μm) (**E**) Heart weight/body weight (HW/BW) ratio. (**F**) Quantification of fibrotic area from Masson’s staining. (**G**) Collagen III protein expression assessed by Western blotting and densitometric quantification normalized to GAPDH. (**H**) Quantification of cardiomyocyte cross-sectional area from WGA staining. (**I**) Serum CK-MB levels. Data are shown as the mean ± SD. n = 5 per group. * *p* < 0.05, ** *p* < 0.01, *** *p* < 0.001.

To investigate the effects of EMPA on DCM mice, a DCM mouse model was established using db/db mice. During the experiment, FBG, food intake, and water consumption were recorded every two weeks. At the end of the experiment, the general condition and cardiac function of these groups were documented (Figure 1A and Figure S2A). The DM group exhibited significant diabetic symptoms, including elevated blood glucose, polydipsia, polyphagia, decreased glucose tolerance and insulin resistance (Figure 1B and Figure S2B–E). The echocardiographic results demonstrated that the EF and FS in DM mice were nearly 30% lower compared to control mice (Figure 1C). In contrast, mice treated with EMPA showed restored diabetic symptoms and cardiac systolic function.

Histological analyses revealed increased heart size, myocardial fibrosis, and hypertrophy in the DM group, which were alleviated by EMPA (Figure 1D). Heart weight/body weight (HW/BW) ratio, fibrosis area, Collagen III expression and cardiomyocyte cross-sectional area were significantly elevated in DM mice and reduced by EMPA, respectively (Figure 1E–H). Serum CK-MB levels were also increased in the DM group and significantly decreased after EMPA treatment (Figure 1I).

### 3.2. Empagliflozin Ameliorates the Mitochondrial Damage and Ferroptosis in DCM Mice

**Figure 2:**
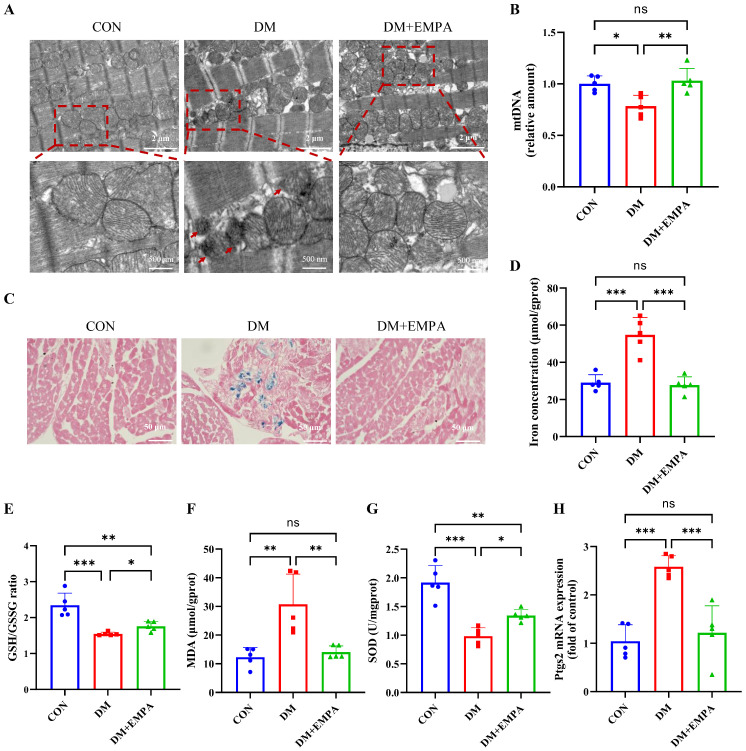
Empagliflozin attenuates ferroptosis in the heart of diabetic mice. (**A**) Representative TEM images showing the mitochondria of cardiac tissue (Scale bars = 2 μm, 500 nm). Ferroptosis is characterized by shrunken mitochondria with increased membrane density or outer membrane rupture (red arrows). (**B**) Relative mtDNA content. (**C**) Representative images of iron staining (Scale bars = 50 μm) (**D**) Iron concentration, (**E**) GSH/GSSH ratio, (**F**) MDA and (**G**) SOD in cardiac tissue lysates were measured from the three groups of mice. (**H**) Relative mRNA levels of Ptgs2. Data are shown as the mean ± SD. n = 5 per group. * *p* < 0.05, ** *p* < 0.01, *** *p* < 0.001.

Transmission electron microscopy revealed severe mitochondrial damage in diabetic myocardium, including cristae disruption, shrinkage, and increased membrane density, consistent with ferroptosis-associated ultrastructural changes; these abnormalities were markedly improved by EMPA (Figure 2A). Mitochondrial DNA content was significantly reduced in the DM group and partially restored in the DM + EMPA group (Figure 2B). Iron staining and total iron concentration showed increased myocardial iron deposition in diabetic mice, which was significantly decreased by EMPA (Figure 2C,D). Diabetic mice exhibited reduced GSH/GSSG ratio and SOD activity and elevated MDA levels, indicating enhanced oxidative stress. These were significantly improved with EMPA treatment (Figure 2E–G). Furthermore, Ptgs2 mRNA expression, a key marker of ferroptosis induced by lipid peroxidation [38,39], was significantly upregulated in the DM group and suppressed by EMPA (Figure 2H). These findings suggest that EMPA ameliorates mitochondrial injury and inhibits ferroptosis in diabetic myocardium.

### 3.3. Empagliflozin Attenuates HG/PA-Induced Cell Death and Oxidative Stress

**Figure 3:**
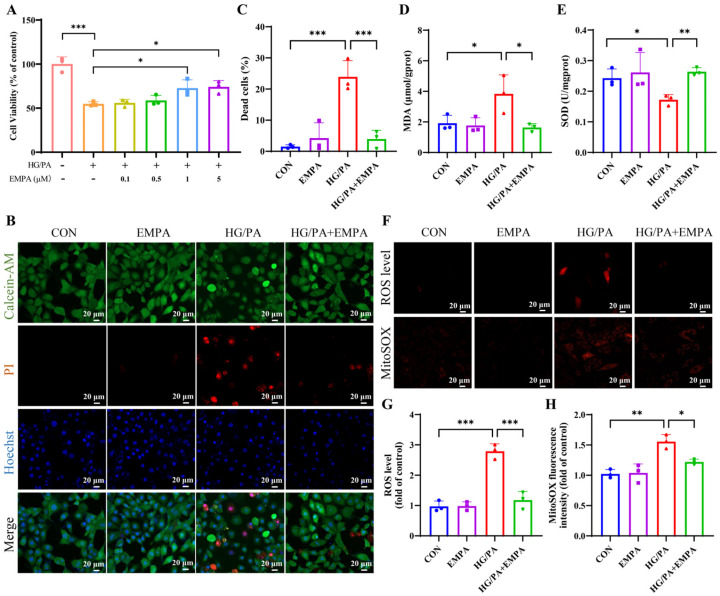
Empagliflozin protects against HG/PA-induced oxidative stress in H9c2 cells. (**A**) Cell viability was assessed following HG/PA treatment with or without different concentrations of EMPA. (**B**) Representative images of live/dead staining using Calcein-AM (green), PI (red), and Hoechst (blue). (**C**) Quantification of dead cells based on PI-positive staining. (**D**) MDA levels and (**E**) the activity of SOD in each group. (**F**) Intracellular and mitochondrial ROS levels were assessed using DHE and MitoSOX probes, respectively. Quantification of fluorescence intensity from (**G**) total ROS and (**H**) mitoROS. Three independent experiments are performed in each study. Data are shown as the mean ± SD. * *p* < 0.05, ** *p* < 0.01, *** *p* < 0.001.

We first treated H9c2 cells with 33.3 mM glucose and 200 μM PA to simulate the HG/PA conditions found in DCM. HG/PA treatment significantly decreased cell viability and increased cell death in H9c2 cells. EMPA dose-dependently improved cell viability and reduced HG/PA-induced cytotoxicity (Figure 3A–C). When the concentration exceeded 1 μM, no further improvement in viability was observed. Therefore, 1 μM was selected as the optimal concentration for subsequent experiments. EMPA significantly attenuated the HG/PA-induced oxidative stress, as evidenced by decreased MDA accumulation and restored SOD activity (Figure 3D,E). ROS and mitochondrial superoxide levels were markedly increased by HG/PA, as detected by DHE and MitoSOX fluorescence, and both were significantly suppressed by EMPA co-treatment (Figure 3F–H).

### 3.4. Empagliflozin Mitigates HG/PA-Induced Ferroptosis in H9c2 Cells

To further confirm the involvement of ferroptosis, we used Fer-1, a selective ferroptosis inhibitor. Figure S3 shows that Fer-1 treatment significantly reduced the levels of total iron, Fe^2+^, MDA, ROS, and mitoROS, and restored the activity of SOD, GSH/GSSG ratio, and Ptgs2 mRNA expression in HG/PA-treated H9c2 cells. These findings suggest that HG/PA induces ferroptosis in H9c2 cells.

**Figure 4:**
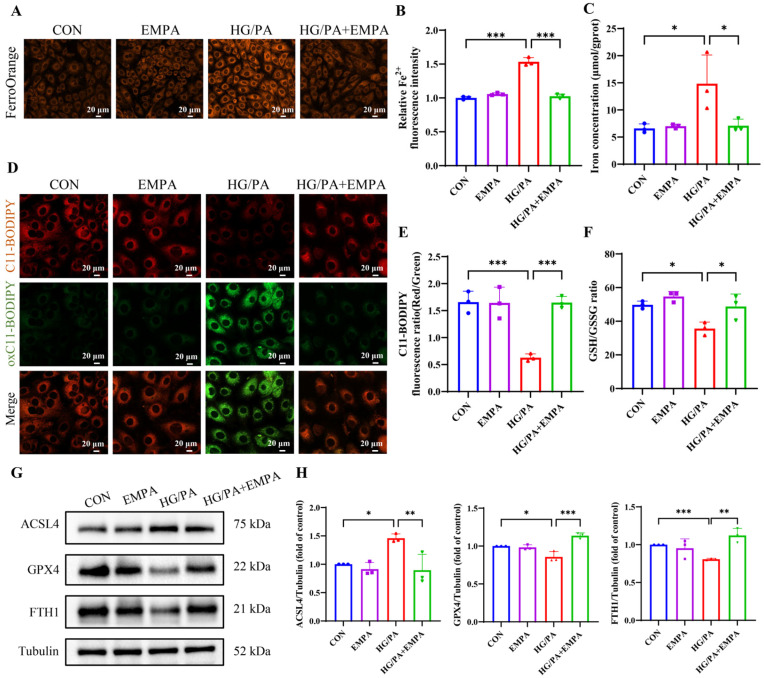
Empagliflozin attenuates HG/PA-induced ferroptosis in H9c2 cells. (**A**) Representative images of FerroOrange fluorescence. (**B**) Quantification of intracellular Fe^2+^ levels. (**C**) Total iron concentration in cells. (**D**) Representative images of lipid peroxidation using C11-BODIPY staining. (**E**) Quantification of red/green fluorescence ratio. (**F**) GSH/GSSG ratio. (**G**) Representative Western blot and (**H**) quantification of ferroptosis-related proteins ACSL4, GPX4, and FTH1. Three independent experiments are performed in each study. Data are shown as the mean ± SD. * *p* < 0.05, ** *p* < 0.01, *** *p* < 0.001.

To explore the anti-ferroptotic effect of EMPA in vitro, ferroptosis markers were examined in H9c2 cells under HG/PA. HG/PA significantly increased Fe^2+^ levels (Figure 4A,B), total iron content (Figure 4C), and decreased FTH1 expression (Figure 4G,H), which were reversed by EMPA. C11-BODIPY staining showed that the level of oxC11-BODIPY was significantly increased in the HG/PA group compared with the CON group, and this effect was reversed by EMPA treatment (Figure 4D,E). The protein expression level of ACSL4 was upregulated after HG/PA treatment and downregulated by EMPA, while GPX4 and GSH/GSSH ratio, suppressed by HG/PA, were restored with EMPA treatment (Figure 4F–H). These results indicate that EMPA alleviates ferroptosis by regulating iron metabolism, lipid peroxidation, and the GSH/GPX4 axis.

### 3.5. Empagliflozin Ameliorates HG/PA-Induced Mitochondrial Dysfunction in H9c2 Cells

**Figure 5:**
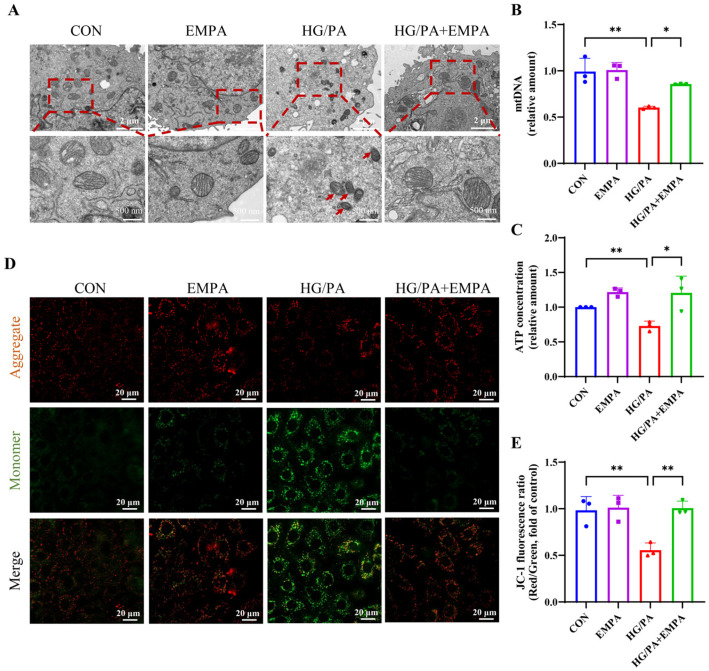
Empagliflozin alleviates HG/PA-induced mitochondrial dysfunction in H9c2 cells. (**A**) Mitochondrial morphology observed by transmission electron microscopy in H9c2 cells. Red arrows indicate mitochondrial alterations indicative of ferroptotic features. (**B**) mtDNA copy number in H9c2 cells. (**C**) ATP levels in H9c2 cells. (**D**) Representative images of mitochondrial membrane potential analyzed by JC-1 staining. (**E**) Quantification of JC-1 fluorescence ratio. Three independent experiments are performed in each study. Data are shown as the mean ± SD. * *p* < 0.05, ** *p* < 0.01.

Mitochondria play a central role in energy metabolism and ROS production, serving as a key site for ferroptosis in cardiomyocytes. TEM revealed that HG/PA caused mitochondrial damage in H9c2 cells, characterized by increased membrane density, mitochondrial shrinkage, and loss of cristae—ultrastructural changes analogous to those observed in the hearts of DCM mice. These morphological abnormalities were partially alleviated by EMPA (Figure 5A). Consistently, HG/PA significantly reduced the copy number of mtDNA, ATP production and MMP, while all of which were restored following EMPA treatment (Figure 5B–E). These findings indicate that EMPA exerts a protective effect on mitochondria.

### 3.6. SIRT3 Is a Potential Target for Empagliflozin

**Figure 6:**
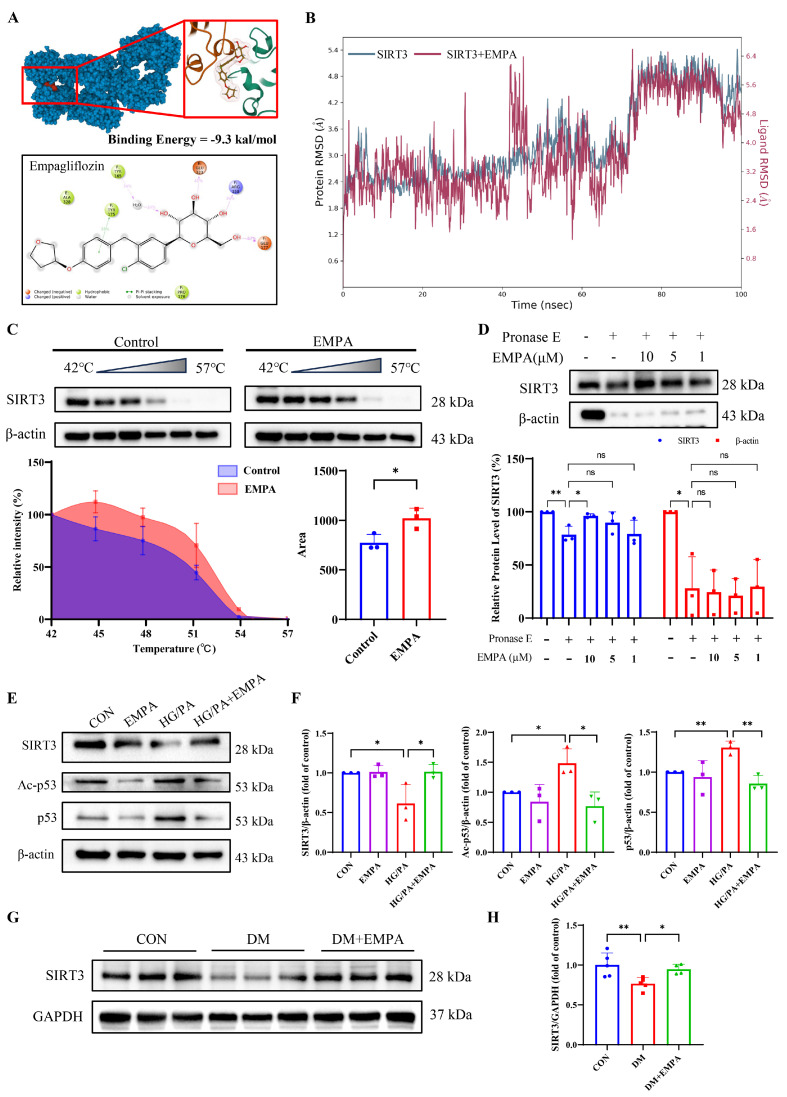
Empagliflozin binds to SIRT3 and enhances its stability. (**A**) Molecular docking diagram of EMPA with SIRT3, including binding energy and binding sites. (**B**) During a 100 ns molecular dynamics simulation, the RMSD of the EMPA–SIRT3 complex remained stable, indicating conformational stability. (**C**) The cellular thermal shift assay was conducted using H9c2 cells in the presence of EMPA (1 μM). The stability of SIRT3 protein under 42–57 °C were measured by Western Blot and quantified by band intensity and area under the curve. (**D**) Cell lysate was incubated with EMPA (0, 1, 5, and 10 μM) and then subjected to Pronase E digestion. The protein level of SIRT3 was measured by Western Blot and quantified by the Image J software. (**E**) Western blot analysis and (**F**) quantification of SIRT3, p53, and ac-p53 protein levels in H9c2 cells. (**G**) Western blot analysis and (**H**) quantification of SIRT3 protein levels in myocardial tissue. Three independent experiments are performed in each study. Data are shown as the mean ± SD. * *p* < 0.05; ** *p* < 0.01; ns, not significant.

SIRT3, which is highly expressed in cardiomyocytes, is crucial for maintaining redox balance and mitochondrial homeostasis [40]. To assess the interaction between EMPA and SIRT3, molecular docking was performed using AutoDock Vina. EMPA showed strong binding affinity to key residues of SIRT3 (ARG158, TYR165, TYR175, GLU177, GLU323) with a binding energy of −9.31 kcal/mol (Figure 6A), indicating spontaneous and strong binding [41]. Molecular dynamics simulations demonstrated a stable binding conformation, which was further stabilized over time (Figure 6B). Additionally, thermal shift assays showed enhanced thermal stability of SIRT3 in the presence of EMPA (Figure 6C). DARTS analysis confirmed that EMPA increased SIRT3 resistance to protease digestion (Figure 6D).

Compared with the control group, SIRT3 levels were significantly decreased in the HG/PA-treated cells, whereas EMPA treatment significantly increased SIRT3 expression (Figure 6E,F). Additionally, p53 is a key downstream target of SIRT3, and SIRT3-mediated deacetylation of p53 plays a crucial role in inhibiting ferroptosis in cardiomyocytes [42]. EMPA suppressed the elevated expression of p53 and its acetylated form (ac-p53) induced by HG/PA, suggesting that SIRT3-mediated p53 deacetylation may contribute to the anti-ferroptotic effect of EMPA (Figure 6E,F). A similar trend was observed in the myocardium of diabetic mice, where SIRT3 protein levels were significantly decreased and EMPA reversed this effect (Figure 6G,H).

### 3.7. Overexpression of SIRT3 Alleviates Ferroptosis in H9c2 Cells Under HG/PA

**Figure 7:**
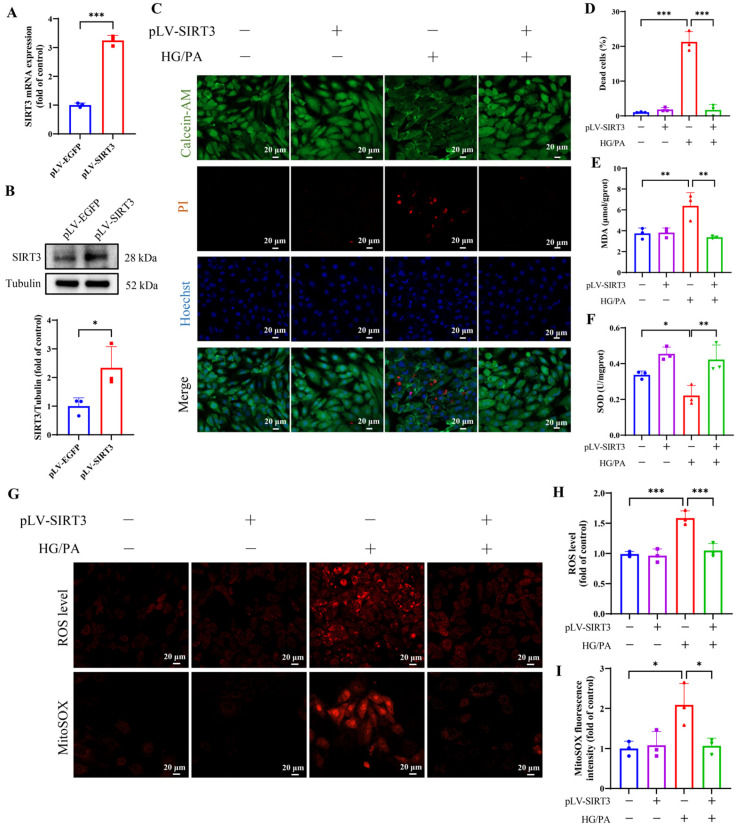
Overexpression of SIRT3 attenuates HG/PA-induced oxidative stress. (**A**) qPCR analysis of SIRT3 mRNA overexpression. (**B**) Representative Western blot and quantification of SIRT3. (**C**) Representative images of live/dead staining using Calcein-AM (green), PI (red), and Hoechst (blue). (**D**) Quantification of dead cells based on PI-positive staining. (**E**) MDA levels. (**F**) SOD activity. (**G**) Intracellular and mitochondrial ROS levels were assessed using DHE and MitoSOX probes, respectively. Quantification of fluorescence intensity from (**H**) total ROS and (**I**) mitoROS. Three independent experiments are performed in each study. Data are shown as the mean ± SD. * *p* < 0.05, ** *p* < 0.01, *** *p* < 0.001.

To investigate the effect of SIRT3 on oxidative stress and ferroptosis in cardiomyocytes under HG/PA, we established SIRT3-overexpressing H9c2 cells via lentiviral infection. qPCR and Western blot confirmed significantly increased SIRT3 mRNA and protein levels in the pLV-SIRT3 group compared to the pLV-EGFP control (Figure 7A,B). SIRT3 overexpression significantly reduced HG/PA-induced cell death (Figure 7C,D). Additionally, MDA levels (Figure 7E), total ROS, and mitoROS (Figure 7G–I) were markedly increased in the HG/PA group, while SOD activity was decreased (Figure 7F). These changes were significantly reversed by SIRT3 overexpression, as evidenced by reduced MDA levels (Figure 7E), increased SOD activity (Figure 7F), and decreased ROS and mitoROS levels (Figure 7G–I), suggesting a protective antioxidant effect.

**Figure 8:**
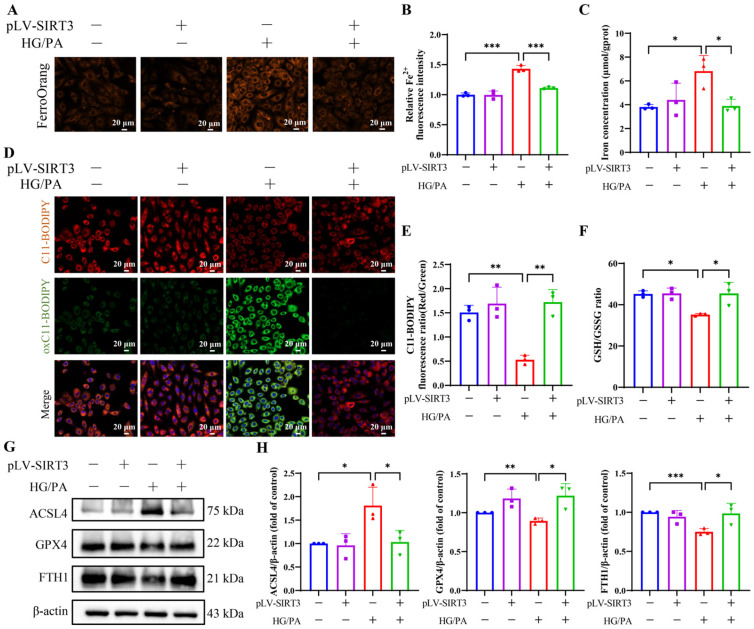
Overexpression of SIRT3 attenuates HG/PA-induced ferroptosis. (**A**) Representative images of FerroOrange fluorescence. (**B**) Quantification of intracellular Fe^2+^ levels. (**C**) Total iron concentration in cells. (**D**) Representative images of lipid peroxidation using C11-BODIPY staining. (**E**) Quantification of red/green fluorescence ratio. (**F**) GSH/GSSG ratio. (**G**) Representative Western blot and (**H**) quantification of ferroptosis-related proteins ACSL4, GPX4, and FTH1. Three independent experiments are performed in each study. Data are shown as the mean ± SD. * *p* < 0.05, ** *p* < 0.01, *** *p* < 0.001.

To assess the impact of SIRT3 on ferroptosis, we further evaluated iron metabolism and lipid peroxidation markers. HG/PA treatment significantly increased intracellular Fe^2+^ levels (Figure 8A,B), total iron (Figure 8C), and decreased FTH1 expression (Figure 8G,H). SIRT3 overexpression reversed these changes, reducing iron accumulation. Lipid peroxidation was also elevated under HG/PA conditions, indicated by increased oxC11-BODIPY fluorescence (Figure 8D,E) and upregulation of ACSL4 (Figure 8G,H); both were significantly reduced following SIRT3 overexpression. Moreover, the HG/PA-induced suppression of GSH/GSSG ratio (Figure 8F) and GPX4 (Figure 8G,H) was significantly rescued by SIRT3 overexpression, further confirming the inhibitory effect on ferroptosis. These findings suggest that SIRT3 attenuates ferroptosis under HG/PA stress by modulating iron metabolism, reducing lipid peroxidation, and enhancing the antioxidant defense system.

### 3.8. SIRT3 Knockdown Alleviated the Protective Effect of Empagliflozin Treatment on Ferroptosis in H9c2 Cells

**Figure 9:**
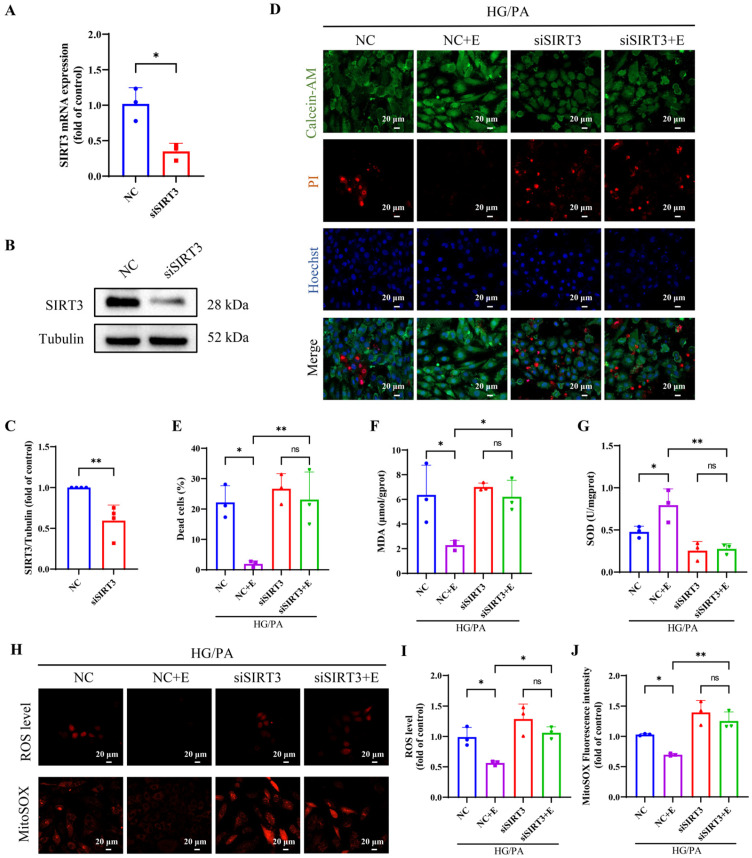
Empagliflozin ameliorates oxidative stress in H9c2 cells induced by HG/PA via SIRT3. (**A**) qPCR analysis of SIRT3 knockdown efficiency at the mRNA level. (**B**) Representative Western blot and (**C**) quantification of SIRT3. (**D**) Representative images of live/dead staining using Calcein-AM (green), PI (red), and Hoechst (blue). (**E**) Quantification of dead cells based on PI-positive staining. (**F**) MDA levels. (**G**) SOD activity. (**H**) Intracellular and mitochondrial ROS levels were assessed using DHE and MitoSOX probes, respectively. Quantification of fluorescence intensity from (**I**) total ROS and (**J**) mitoROS. Three independent experiments are performed in each study. Data are shown as the mean ± SD. * *p* < 0.05; ** *p* < 0.01; ns, not significant.

To determine whether EMPA attenuates ferroptosis through the SIRT3 pathway under HG/PA, we transfected H9c2 cells with SIRT3 siRNA. Compared to the NC group, SIRT3 mRNA and protein expression were significantly decreased in the siSIRT3 group (Figure 9A–C). Calcein-AM/PI staining showed that EMPA significantly reduced cell death in the HG/PA+NC+E group compared to HG/PA + NC group, an effect that was abrogated by SIRT3 knockdown (Figure 9D,E). Similarly, EMPA reduced MDA levels (Figure 9F), total ROS, and mitoROS levels (Figure 9H–J), while enhancing SOD activity (Figure 9G). These antioxidant effects were blocked by SIRT3 silencing.

**Figure 10:**
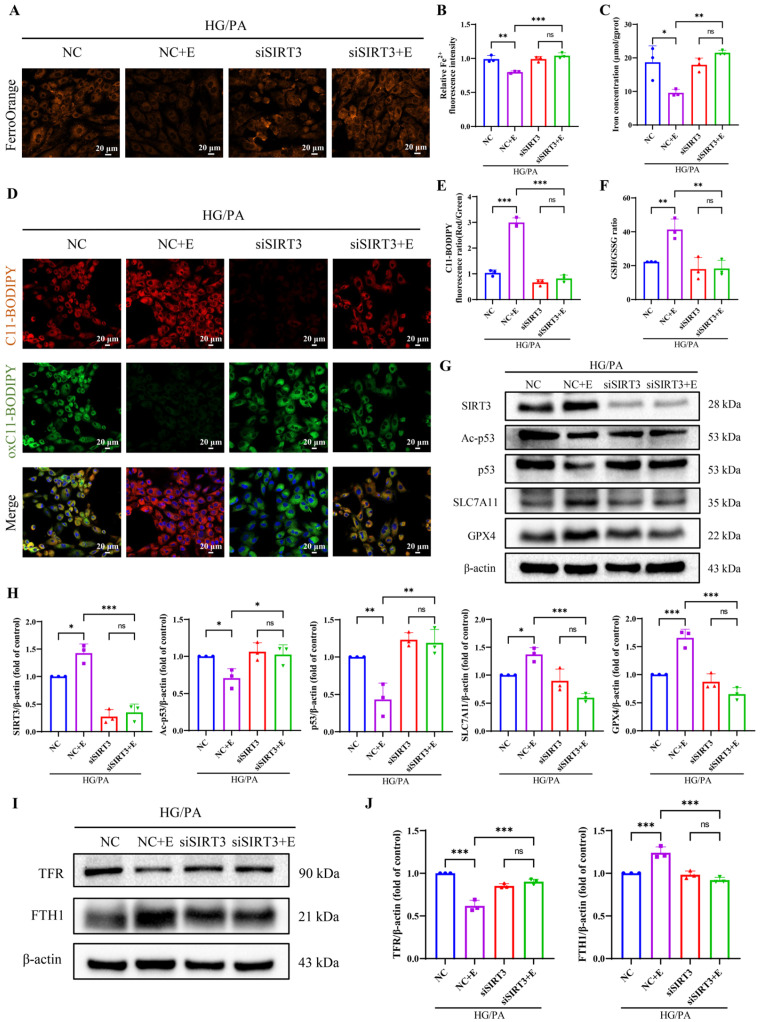
Empagliflozin ameliorates ferroptosis in H9c2 cells induced by HG/PA via SIRT3. (**A**) Representative images of FerroOrange fluorescence. (**B**) Quantification of intracellular Fe^2+^ levels. (**C**) Total iron concentration in cells. (**D**) Representative images of lipid peroxidation using C11-BODIPY staining. (**E**) Quantification of red/green fluorescence ratio. (**F**) GSH/GSSG ratio. (**G**) Representative Western blot and (**H**) quantification of SIRT3, ac-p53, p53, SLC7A11 and GPX4 proteins. (**I**) Representative Western blot and (**J**) quantification of TFR and FTH1 proteins. Three independent experiments are performed in each study. Data are shown as the mean ± SD. * *p* < 0.05; ** *p* < 0.01; *** *p* < 0.001; ns, not significant.

To assess ferroptosis, we measured relevant markers. Fe^2+^ levels (Figure 10A,B) and total iron content (Figure 10C) were significantly reduced in the HG/PA + NC + E group compared to HG/PA + NC group, but this reduction was absent in SIRT3-silenced cells. Likewise, EMPA suppressed lipid peroxidation (Figure 10D,E) and restored GSH levels (Figure 10F), effects that were reversed by SIRT3 knockdown. EMPA increased SIRT3 expression in NC-transfected cells, but not in SIRT3-silenced cells (Figure 10G,H). It has been reported that solute carrier family 7 member 11 (SLC7A11) and GPX4 are downstream targets of p53 [36]. Consistently, in our study, EMPA reduced the expression of p53 and ac-p53, while upregulating the SLC7A11/GPX4 axis (Figure 10G,H). These effects were not observed when SIRT3 was knocked down. EMPA was observed to regulate iron metabolism through the reduction of TFR and the elevation of FTH1, with SIRT3 knockdown diminishing these regulatory effects (Figure 10I,J). These findings suggest that EMPA mitigates HG/PA-induced cardiomyocyte ferroptosis through SIRT3 activation, which subsequently modulates both the p53/SLC7A11/GPX4 signaling pathway and iron metabolism.

## 4. Discussion

In the present study, db/db mice (a well-established animal model of T2DM) exhibited characteristic metabolic disturbances including polydipsia, polyphagia, hyperglycemia, hyperlipidemia and insulin resistance, which are consistent with the clinical manifestations of T2DM. To simulate glucolipotoxicity in vitro, H9c2 cardiomyocytes were treated with HG and PA. We systematically evaluated the effects of EMPA on both db/db mice and HG/PA-treated H9c2 cells. Our findings demonstrate that EMPA confers direct cardioprotection by enhancing SIRT3-mediated inhibition of ferroptosis.

DCM is a diabetes-induced cardiomyopathy independent of traditional cardiovascular risks, characterized by myocardial fibrosis, ventricular remodeling, and contractile dysfunction [43]. Its pathogenesis involves hyperglycemia-induced oxidative stress and lipotoxicity (from excessive fatty acid oxidation), collectively promoting cardiomyocyte death [14,21,44]. Consistent with these mechanisms, our study demonstrated that db/db mice developed DCM accompanied by hyperglycemia-induced oxidative stress, mitochondrial damage, and increased cardiomyocyte ferroptosis, both in vivo and in vitro.

While some oral glucose-lowering agents have been associated with an increased risk of life-threatening arrhythmias in patients with T2DM [45], EMPA has demonstrated a favorable cardiovascular safety profile. EMPA, as a SGLT2 inhibitor, promotes urinary glucose excretion by inhibiting the reabsorption of glucose in the renal proximal tubules, thereby reducing blood glucose levels. Beyond glycemic control, EMPA exerts pleiotropic protective effects in multiple organs, including the kidney [46] and the heart [47]. Its cardioprotective actions involve improving cardiac energy metabolism and reducing oxidative stress, myocardial fibrosis, and cell death [47]—key factors in DCM progression. Studies have shown that EMPA can reduce the production of reactive ROS, restore antioxidant defense capabilities, and improve mitochondrial function, all of which are essential for maintaining myocardial energy metabolism and contractile function [48]. By alleviating these pathological processes, EMPA helps protect cardiac function and may prevent the deterioration of DCM. In this study, we found that db/db mice developed DCM at 20 weeks of age, a finding supported by the results of Li et al. [33]. EMPA prevented DCM by enhancing the impaired contractile function and reducing myocardial fibrosis in db/db mice. Additionally, similar to previous studies [48], we observed that EMPA also reduced hyperglycemia and insulin resistance in db/db mice, while lowering oxidative stress levels and mitochondrial dysfunction in myocardial tissue. Ideally, studies with equivalent glucose levels between db/db and EMPA-treated db/db groups are needed to confirm that EMPA has effects beyond glucose lowering. However, accumulating evidence supports that the cardiovascular benefits of EMPA extend beyond glycemic control. In the EMPA-REG OUTCOME trial, its cardioprotective effects could not be fully explained by antihyperglycemic activity alone [26]. Likewise, Ideishi et al. showed that EMPA preserved cardiac function in diabetic mice independent of blood glucose levels [49]. PA, as a representative long-chain non-esterified saturated fatty acid, has strong lipotoxicity to cells. In vitro results showed that treatment with HG and PA significantly increased the mortality of H9c2 cells and enhanced oxidative stress and mitochondrial dysfunction. However, EMPA treatment significantly inhibited HG/PA-induced damage in H9c2 cells. It should be noted that the mechanism by which EMPA acts directly on cardiomyocytes via SGLT2 remains controversial. Although SGLT2 expression has been detected in human cardiomyocytes [27,28], several studies have proposed that SGLT2-independent mechanisms may also exist [50]. We acknowledge that SGLT2 expression was not examined in our mouse hearts or H9c2 cells. Future studies using SGLT2 knockout or knockdown models (in vivo or in H9c2 cells) are needed to determine whether the cardioprotective effects of EMPA observed here are dependent on SGLT2 expression.

Ferroptosis contributes to DCM progression by inducing cardiomyocyte death, leading to contractile dysfunction and cardiac remodeling [43]. This iron-dependent cell death process, implicated in various cardiovascular diseases [51], is driven by oxidative stress and mitochondrial dysfunction in diabetes [52]. Recently discovered mechanisms include: (1) HG-induced p- extracellular-regulated kinase 1/2 activating ferritinophagy [53]; (2) nuclear factor erythroid 2-related factor 2 (NRF2)-GPX4 axis suppression [54]; and (3) impaired mitoGSH/GPX4 pathway [55], suggesting ferroptosis inhibition as a therapeutic target. Several recent studies have implicated SGLT2 inhibitors in the regulation of ferroptosis in DCM. For example, canagliflozin has been shown to ameliorate DCM by inhibiting ferroptosis [14,56]. Research has found that EMPA can regulate iron metabolism and maintain iron homeostasis in the body and the heart [57] and, notably, it suppressed cardiomyocyte ferroptosis in DCM mouse models [58]. The role of ferroptosis in the cardioprotective effects of EMPA is increasingly gaining attention. Considering the antioxidant properties of EMPA, we hypothesize that the protective effects of EMPA on HG/PA-induced injury in H9c2 cardiomyocytes may involve ferroptosis. Our experimental conditions (33.3 mM glucose, 200 μM PA, 24 h) are within the range commonly used to induce ferroptosis in H9c2 cells [13,39,59]. As expected, the ferroptosis inhibitor Fer-1 can eliminate ferroptosis in HG/PA-induced H9c2 cells. EMPA alleviated ferroptosis, oxidative stress, and mitochondrial dysfunction induced by glucolipotoxicity in H9c2 cells, indicating that the protective effects of EMPA on HG/PA-induced H9c2 cells are partly achieved through the inhibition of ferroptosis. Similarly to the streptozocin (STZ) and high fat diet-induced T2DM mouse models [37], we also observed the occurrence of ferroptosis in the myocardial tissue of db/db mice, characterized by decreased SOD activity and GSH/GSSG ratio, and significantly increased iron content, MDA, and Ptgs2 mRNA levels. Moreover, TEM of the cardiac tissue in DCM mice further suggested characteristic mitochondrial changes in ferroptosis. EMPA treatment inhibited ferroptosis in DCM. These findings provide reliable evidence for the inhibitory effects of EMPA on ferroptosis in DCM. Nevertheless, it is worth noting that other forms of cell death, such as apoptosis, have also been detected under similar experimental conditions [39,59]. Although we did not assess apoptosis in the present study, the potential interplay between ferroptosis and apoptosis in the cardioprotective effects of EMPA warrants further investigation.

SIRT3 is a deacetylase that is localized in the mitochondria and regulates cellular metabolism by deacetylating a variety of protein substrates [60]. Research has found that SIRT3 expression is significantly reduced in the hearts of DCM mice, while activation of SIRT3 can improve cardiac function [24]. SIRT3-deficient mice exhibit exacerbated metabolic syndrome after STZ induction, such as accelerated insulin resistance, obesity, hyperlipidemia, and non-alcoholic steatohepatitis [61]. The antioxidant effects of SIRT3 in cardiomyocytes induced by HG have also been confirmed [62]. SIRT3 inhibits ferroptosis through multiple mechanisms, including enhancing antioxidant defenses and improving mitochondrial function [63,64]. Previous studies have found that in DCM model mice, Sentrin/small ubiquitin-like modifier (SUMO)-specific protease 1 can enhance mitochondrial functional stability and reduce cardiomyocyte ferroptosis by promoting the desumoylation of SIRT3, thereby improving DCM [65]. Interestingly, Luo et al. found that EMPA can upregulate the expression of SIRT3 in cardiomyocyte mitochondria [66]. SIRT3 also plays a crucial role in maintaining iron homeostasis by regulating key metabolic pathways that restrict intracellular Fe^2+^, reducing ROS generation and lowering substrate availability for lipid peroxidation, and alleviating ferroptosis in cardiomyocytes [67]. Similarly to the above findings, SIRT3 expression is significantly decreased in DCM model mice and cardiomyocytes induced by HG/PA; whereas, EMPA intervention markedly restored SIRT3 expression. Additionally, overexpression of SIRT3 can reverse cell ferroptosis induced by high fat, while silencing of SIRT3 can eliminate the effects of EMPA treatment on HG/PA-induced death, oxidative stress, mitochondrial dysfunction, and ferroptosis in H9c2 cardiomyocytes.

The p53 protein is widely regarded as the “guardian of the genome” due to its crucial role in maintaining genomic stability [68]. As a key regulator of cellular processes, p53 is involved in a variety of cellular activities, such as cell differentiation, senescence, proliferation, apoptosis, ferroptosis, and autophagy [68]. Evidence has shown that SIRT3 can activate the deacetylation of p53 in H9c2 cells to alleviate ferroptosis-induced cardiomyocyte death caused by myocardial inflammation [69]. The acetylation of p53 protein regulates its stability and conformational changes, thereby promoting p53 activation [70]. Specifically, the C-terminal lysine residues of p53 can be acetylated, as well as ubiquitinated by murine double minute 2 (MDM2). Since acetylation and ubiquitination compete for the same sites, the acetylation of p53 not only occupies the ubiquitination sites of acetylated lysine residues but also weakens the ubiquitination of other unacetylated lysine residues mediated by MDM2 by inducing conformational changes in the protein [70]. Previous studies have shown that the deacetylation of p53 can significantly mitigate the pathological process of STZ-induced DCM by inhibiting the ferroptosis pathway [71]. p53 inhibits SLC7A11 expression, which blocks cellular cystine uptake and inactivates System Xc−. This suppresses the synthesis of GPX4 substrates, leading to reduced cellular antioxidant capacity and GPX4 activity [42]. Based on the above studies, we hypothesize that EMPA treatment activates SIRT3, which mediates the deacetylation of p53. We found that overexpression of SIRT3 can inhibit the acetylation level of p53 in H9c2 cells. In addition, EMPA treatment significantly attenuated the increase in p53 expression and acetylation levels induced by HG/PA in H9c2 cells and enhanced the expression of SLC7A11 and GPX4, which was essentially reversed by SIRT3 silencing. This further indicates that EMPA helps to deacetylate p53 in H9c2 cells treated with HG/PA by enhancing SIRT3 expression.

Notably, a recent study reported that EMPA inhibits cardiomyocyte ferroptosis via the ubiquitin-specific protease 7/NRF2 signaling pathway [58]. Given that NRF2 activates antioxidant gene expression and may interact with SIRT3 [72], we speculate that in addition to the SIRT3-p53 axis, SIRT3 could function downstream of or in parallel with NRF2 to mediate the anti-ferroptotic effects of EMPA. Interestingly, crosstalk between p53 and NRF2 pathways has also been reported in diabetic mouse models [73]. However, the precise interplay among these pathways requires further investigation. Alternatively, it remains possible that EMPA protects cardiomyocytes indirectly by ameliorating oxidative stress, rather than through direct molecular binding. This study still has certain limitations. First, in vivo SIRT3 knockout/knockdown models and a therapeutic (rather than preventive) intervention strategy would improve clinical translatability. Second, the in silico docking prediction requires experimental validation (e.g., IP or pull-down), and a more comprehensive assessment—including additional ferroptosis markers (e.g., ferritin light chain or nuclear receptor coactivator 4) and apoptotic indicators—would provide a fuller characterization of cell death. Future studies are warranted to address these issues.

## 5. Conclusions

**Figure 11:**
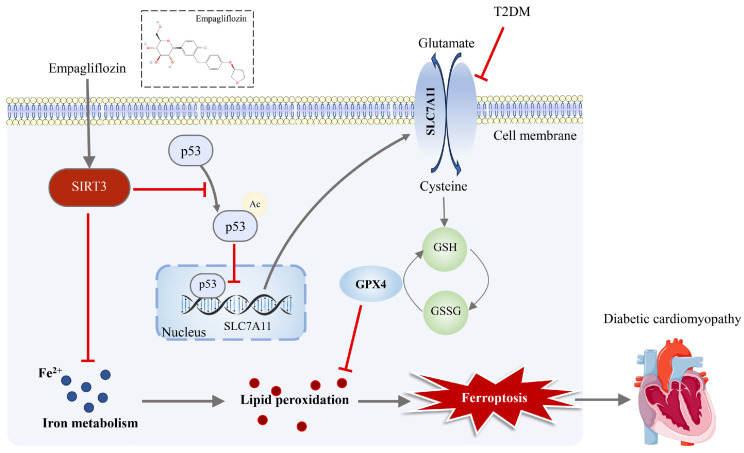
Schematic Illustration of the Mechanism by Which Empagliflozin Inhibits Ferroptosis and Ameliorates Diabetic Cardiomyopathy via the SIRT3-p53/SLC7A11/GPX4 Pathway. T2DM inhibits SLC7A11, promoting ferroptosis and contributing to DCM. EMPA activates SIRT3, which deacetylates and suppresses p53, thereby upregulating SLC7A11 and enhancing GSH synthesis. This reduces lipid peroxidation and ferroptosis via GPX4. SIRT3 also modulates iron metabolism.

In summary, our research indicates that the ferroptosis-inhibiting effect of EMPA on DCM is dependent on SIRT3. EMPA not only suppresses the blood glucose fluctuations in db/db mice but also ameliorates cardiac dysfunction, mitochondrial damage, and myocardial ferroptosis. Additionally, it has been demonstrated that the alleviation of HG/PA-induced cell death, oxidative stress, mitochondrial dysfunction, and ferroptosis in H9c2 cells mediated by EMPA is closely related to SIRT3-dependent deacetylation of p53 (Figure 11). This study is the first to demonstrate that EMPA exerts a protective effect on cardiomyocytes by inhibiting ferroptosis and highlights the significance of EMPA in inhibiting ferroptosis through the activation of SIRT3. The identification of the SIRT3–ferroptosis axis as a previously unrecognized mechanism of EMPA highlights the novelty of our findings and provides a novel perspective for the application of EMPA in the treatment of DCM.

## Data Availability

The original contributions presented in this study are included in the article and Supplementary Materials. Further inquiries can be directed to the corresponding authors.

## Supplementary Materials

The following supporting information can be downloaded at: https://www.mdpi.com/article/10.3390/antiox15050543/s1, Figure S1: The recombinant plasmid pLV-rSIRT3 information; Figure S2: Changes in metabolic profiles after the EMPA intervention in db/db mice; Figure S3: HG/PA induces ferroptosis in H9c2 cells; Table S1: Primer Sequences; Table S2: mtDNA-related Primer Sequences.

## Abbreviations

The following abbreviations are used in this manuscript:

ACSL4	Acyl-CoA Synthetase Long-Chain Family Member 4
ATP	Adenosine Triphosphate
CETSA	Cellular Thermal Shift Assay
DARTS	Drug Affinity Responsive Target Stability
DCM	Diabetic Cardiomyopathy
DM	Diabetes Mellitus
EF	Ejection Fraction
EMPA	Empagliflozin
FBG	Fasting Blood Glucose
FS	Fractional Shortening
FTH1	Ferritin Heavy Chain 1
GPX4	Glutathione Peroxidase 4
GSH	Glutathione
GSSG	Glutathione Oxidized
HE	Hematoxylin and Eosin
HG	High Glucose
MDA	Malondialdehyde
MitoROS	Mitochondrial Reactive Oxygen Species
MMP	Mitochondrial Membrane Potential
mtDNA	Mitochondrial DNA
NAD^+^	Nicotinamide Adenine Dinucleotide
NC	Negative Control
PA	Palmitic Acid
PBS	Phosphate-Buffered Saline
Ptgs2	Prostaglandin-Endoperoxide Synthase 2
RMSD	Root Mean Square Deviation
ROS	Reactive Oxygen Species
qPCR	Quantitative Real-time PCR
SGLT2	Sodium-Glucose Cotransporter 2
siRNA	Small Interfering RNA
SIRT3	Silent Information Regulator 3
SLC7A11	Solute Carrier Family 7 Member 11
SOD	Superoxide Dismutase
STZ	Streptozocin
T2DM	Type 2 Diabetes Mellitus
TFR	Transferrin Receptor
WGA	Wheat Germ Agglutinin
