# Supplementary material for: Empagliflozin Ameliorates Diabetic Cardiomyopathy by Inhibiting Ferroptosis via SIRT3: Mechanisms and Therapeutic Implications
Source: Antioxidants (Basel). 2026 Apr 24;15(5):543. doi: 10.3390/antiox15050543 (PMC13203827; doi:10.3390/antiox15050543)
Supplement: Supplementary file 1 [file antioxidants-15-00543-s001.zip › antioxidants-4216021-supplementary.pdf]

Supplementary data

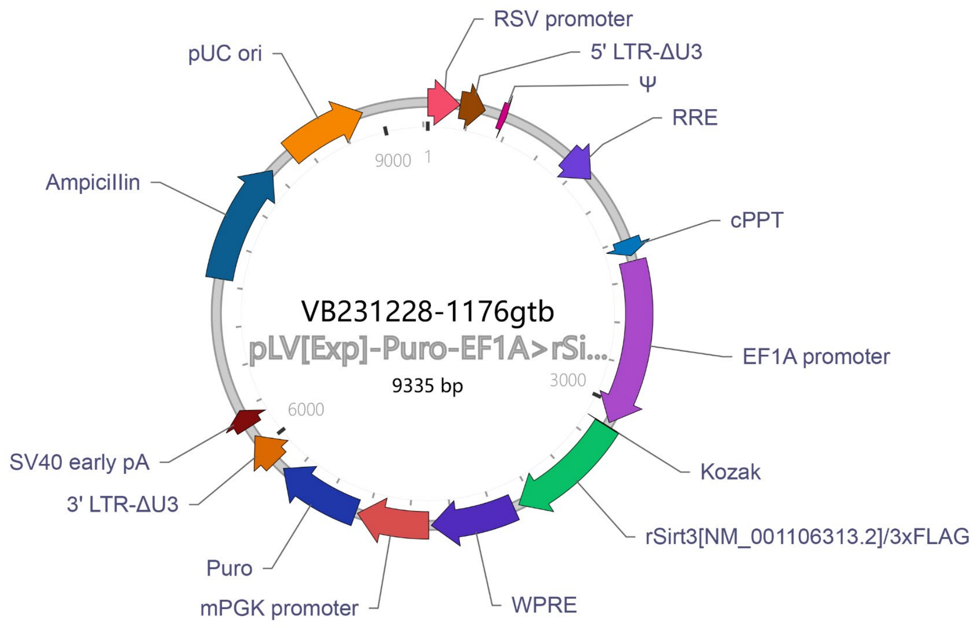

Figure S1. The recombinant plasmid pLV-rSIRT3 information.

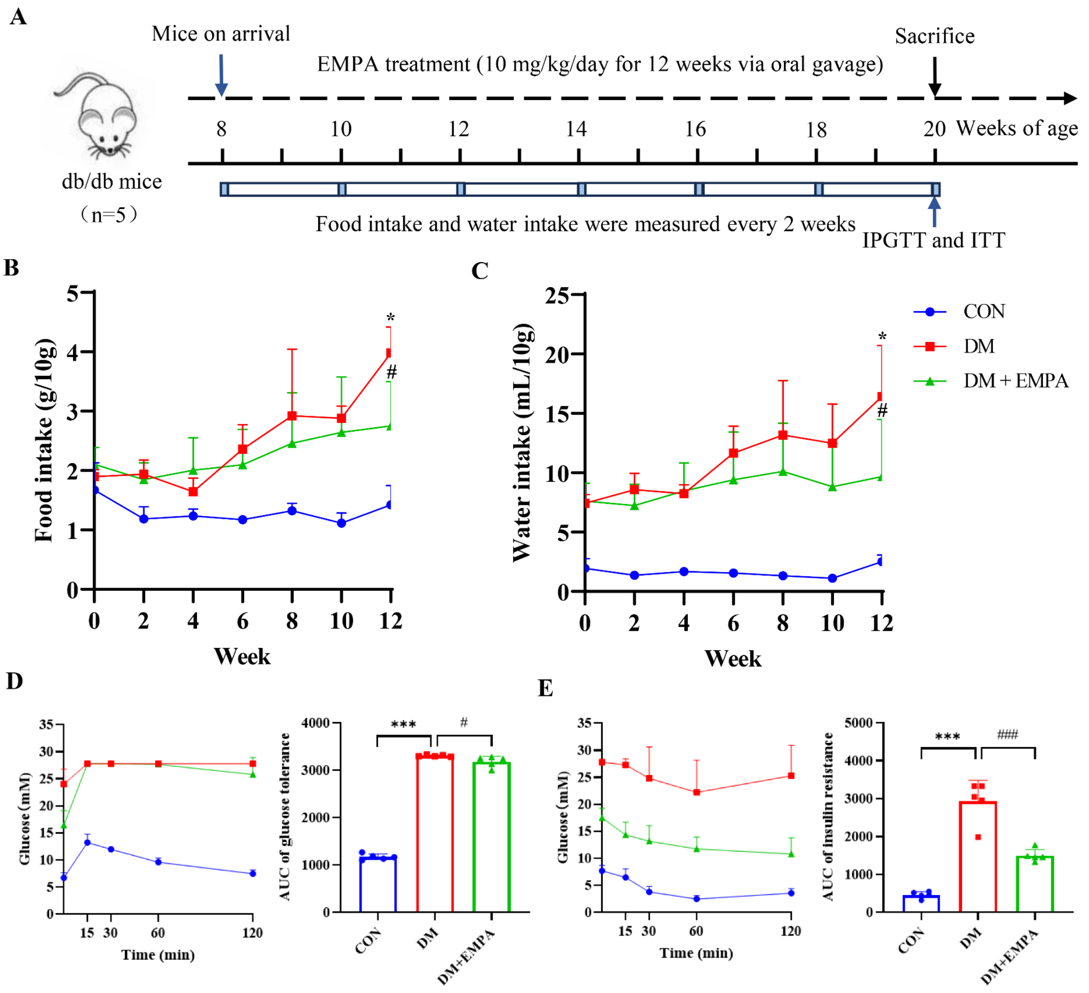

Figure S2. Changes in metabolic profiles after the EMPA intervention in db/db mice. (**A**) Treatment scheme for the DM+EMPA group. (**B-C**) The food, and water intake of the CON, DM, and DM+EMPA groups were measured each two weeks. (**D)** Glucose tolerance. (**E**) Insulin tolerance. Data are shown as the mean ± SD. n = 5 per group. *p < 0.05 , ***p < 0.001 vs CON; $^{\#}p$ < 0.05 , $^{\#\#\#}p$ < 0.001 vs DM.

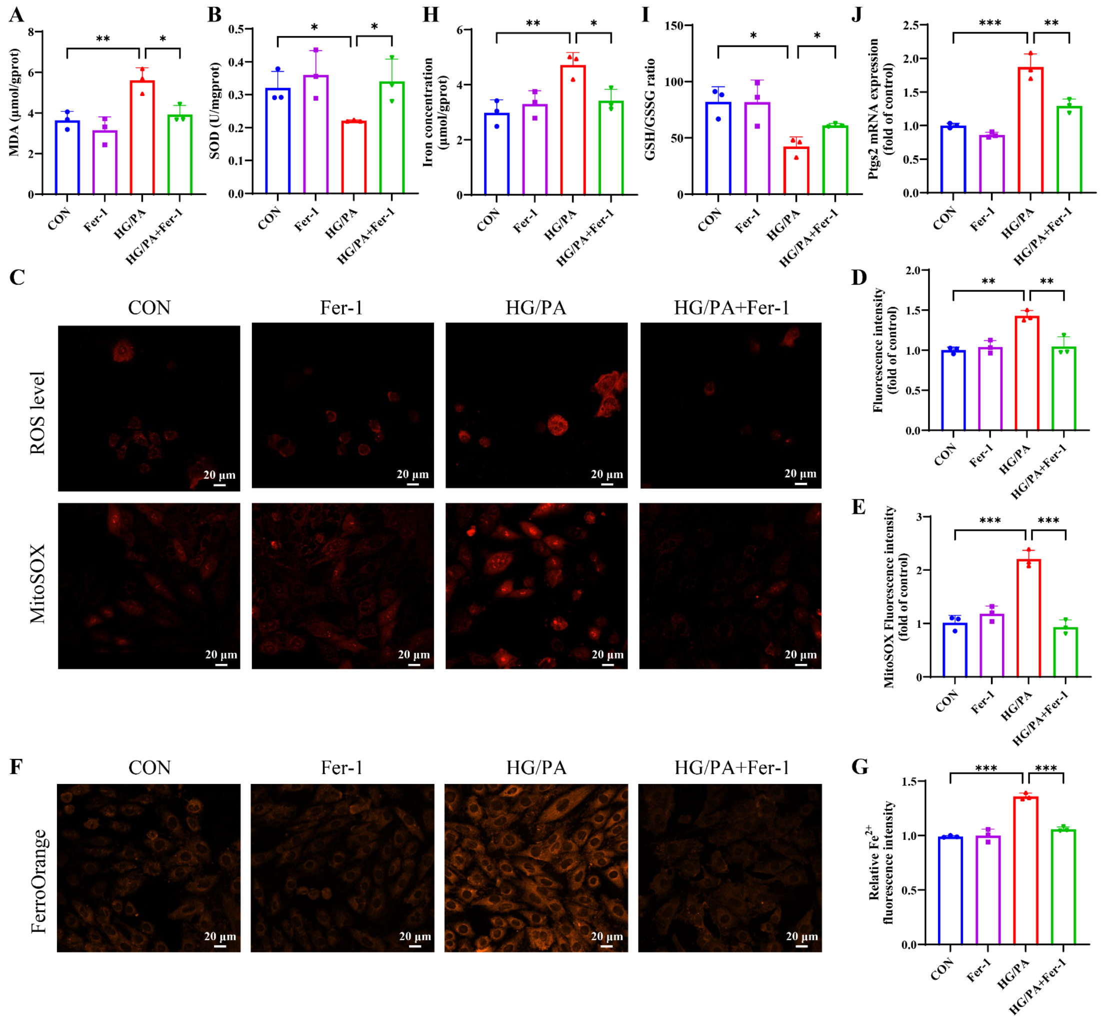

Figure S3. HG/PA induces ferroptosis in H9c2 cells. (**A**) MDA levels. (**B**) SOD activity. **(C)** Intracellular and mitochondrial ROS levels were assessed using DHE and MitoSOX probes, respectively. (**D-E**) Quantification of fluorescence intensity from total ROS and mitoROS. (**F-G**) Representative FerroOrange fluorescence images and quantification of intracellular Fe^{2+} levels. (**H**) Total iron concentration. (**I**) GSH/GSSG ratio. (**J**) Relative mRNA levels of Ptgs2. Three independent experiments are performed in each study. Data are shown as the mean ± SD. **p* < 0.05, ***p* < 0.01, ****p* < 0.01.

Table S1. Primer Sequences

Gene	Organism	Sequence (5’→3’)
Ptgs2	Mouse	F: TGAGCAACTATTCCAAACCAGC<br>R: GCACGTAGTCTTCGATCACTATC
Actb	Mouse	F: GGCTGTATTCCCCTCCATCG<br>R: CCAGTTGGTAACAATGCCATGT
Sirt3	Rat	F: ACTACTTCCTTCGGCTGCTTCAC<br>R: CAGGGATACCAGATGCTCTCTCAAG
Ptgs2	Rat	F: ATGTTCGCATTCTTTGCCCAG<br>R: TACACCTCTCCACCGATGAC
Actb	Rat	F: TCGTGCGTGACATTAAAGAG<br>R: ATTGCCGATAGTGATGACCT

F, Forward primer; R, Reverse primer.

Table S2. mtDNA-related Primer Sequences

Gene	Organism	Sequence (5’→3’)
COX II	Mouse	F：TCATAATTGCCCTCCCCTCTC<br>R：ACTTCTAGCAGTCGTAGTTCACCAG
Rps18	Mouse	F：AGTTCCAGCACATTTTGCGAG<br>R：TCATCCTCCGTGAGTTCTCCA
ND-1	Rat	F：AAGCGGCTCCTTCTCCCTACAAAT<br>R：GAAGGGAGCTCGATTTGTTTCTGC
β -actin	Rat	F：TGAGAGGGAAATCGTGCGTGAC<br>R：GGAAGAGGATGCGGCAGTGG

F, Forward primer; R, Reverse primer.
